# Multidrug-Resistant Gram-Negative Bacteria and Extended-Spectrum β-Lactamase-Producing Klebsiella pneumoniae from the Poultry Farm Environment

**DOI:** 10.1128/spectrum.02694-21

**Published:** 2022-04-25

**Authors:** Yuvaneswary Veloo, Syahidiah S. A. Thahir, Sakshaleni Rajendiran, Lim K. Hock, Norazah Ahmad, Vickneshwaran Muthu, Rafiza Shaharudin

**Affiliations:** a Institute for Medical Research, National Institutes of Health, Ministry of Health, Setia Alam, Selangor, Malaysia; b Disease Control Division, Ministry of Health, Putrajaya, Malaysia; Institut Pasteur

**Keywords:** poultry, antibiotics, multidrug resistance, Gram-negative bacteria, ESBL

## Abstract

The indiscriminate use and overuse of various antibiotics have caused the rapid emergence of antibiotic-resistant bacteria (ARB) in poultry products and the surrounding environment, giving rise to global public health issues. This study aimed to determine the prevalence of multidrug-resistant (MDR) Gram-negative bacteria (GNB) found in the environment of poultry farms and to evaluate the risk of contamination in these farms based on multiple antibiotic resistance (MAR) index values. Soil and effluent samples were collected from 13 poultry farms. The VITEK 2 system was used for bacterial identification and susceptibility testing of the isolates. The identified Gram-negative isolates were Acinetobacter spp., *Aeromonas* spp., Enterobacter spp., Klebsiella pneumoniae, Proteus spp., *Providencia* spp., Pseudomonas spp., and Sphingomonas paucimobilis. The results showed that Enterobacter spp.*, Aeromonas* spp., and *Providencia* spp. exhibited the highest MDR rates and MAR indices; 14% of K. pneumoniae isolates (3/21 isolates) were resistant to 13 antibiotics and found to be extended-spectrum β-lactamase (ESBL)-producing bacteria. As for the tested antibiotics, 96.6% of the isolates (28/29 isolates) demonstrated resistance to ampicillin, followed by ampicillin-sulbactam (55.9% [33/59 isolates]) and cefazolin (54.8% [57/104 isolates]). The high percentage of MDR bacteria and the presence of ESBL-producing K. pneumoniae strains suggested the presence of MDR genes from the poultry farm environment, which poses an alarming threat to the effectiveness of the available antibiotic medicines to treat infectious diseases. Therefore, the use of antibiotics should be regulated and controlled, while studies addressing One Health issues are vital for combating and preventing the development and spread of ARB.

**IMPORTANCE** The occurrence and spread of ARB due to high demand in poultry industries are of great public health concern. The widespread emergence of antibiotic resistance, particularly MDR among bacterial pathogens, poses challenges in clinical treatment. Some pathogens are now virtually untreatable with current antibiotics. However, those pathogens were rarely explored in the environment. In alignment with the concept of One Health, it is imperative to study the rate of resistance in the environment, because this domain plays an important role in the dissemination of bacteria to humans, animals, and other environmental areas. Reliable data on the prevalence of MDR bacteria are crucial to curb the spread of bacterial pathogens that can cause antimicrobial-resistant infections.

## OBSERVATION

Antibiotic resistance (AR) is recognized as “the silent tsunami facing modern medicine” (1). The occurrence, spread, and persistence of AR is a major global public health concern. The significance of the overuse and misuse of veterinary antibiotics is worrying, because it contributes to the increase in the emergence and spread of antibiotic-resistant bacteria (ARB), causing infections in both humans and animals (2).

Studies have found that Gram-negative bacteria (GNB), particularly *Enterobacteriaceae* strains, are capable of acquiring resistance via plasmid-mediated horizontal transmission of resistance genes (3). Therefore, the extensive usage of antibiotics as feed preservatives and prophylaxis is of concern, because it can lead to the emergence of organisms resistant to the utmost antibiotics. Hence, the World Health Organization (WHO) has introduced a list of antibiotic-resistant priority pathogens that present a great threat to humans; the majority of the pathogens are GNB (4). Since GNB other than Salmonella spp. and Escherichia coli have been less well explored, this study prioritized determining the prevalence of multidrug-resistant (MDR) GNB from poultry farm environments and evaluating the risk of contamination on the farms based on the multiple antibiotic resistance (MAR) indices of the isolates.

A cross-sectional baseline study was carried out from January 2018 to October 2019. A total of 39 soil samples and 39 effluent samples were collected by trained personnel from 13 poultry farms that were registered under the Department of Veterinary Services, Selangor, Malaysia. Isolation and enumeration of bacteria were performed using the spread plate method. The VITEK 2 GN card (bioMérieux, Nürtingen, Germany) was used for bacterial identification, whereas the VITEK 2 AST-GN83 susceptibility card (bioMérieux) was used to determine the MICs of GNB. The MAR index for each bacterial isolate and poultry farm sample was calculated based on the method described by Krumperman (5). MAR index values of <0.2 indicate a low risk of ARB contamination, while values of ≥0.2 indicate a high risk of ARB contamination (5, 6).

A total of 104 isolates were obtained from the environmental (soil and effluent) samples collected from 13 poultry farms (see Table S1 in the supplemental material). Eight GNB that were successfully cultured from these environmental samples were Acinetobacter spp., *Aeromonas* spp., Enterobacter spp., Klebsiella pneumoniae, Proteus spp., *Providencia* spp., Pseudomonas spp., and Sphingomonas paucimobilis. Table 1 shows the susceptibility testing results for each isolate based on the various antibiotics present on the AST-GN83 test card. The Enterobacter isolates had the greatest resistance (29.0%) against the tested antibiotics, followed by the *Aeromonas* isolates (26.7%) and the *Providencia* isolates (23.5%).

**TABLE 1:** Susceptibility testing results for the isolates based on various antibiotics on the AST-GN83 card

Drug	No. of isolates^*a*^															
	Acinetobacter spp.		*Aeromonas* spp.		Enterobacter spp.		K. pneumoniae		Proteus spp.		*Providencia* spp.		Pseudomonas spp.		S. paucimobilis	
	S	R	S	R	S	R	S	R	S	R	S	R	S	R	S	R
AMP	—	—	0	1	—	—	0	21	1	3	0	3	—	—	—	—
AMC	—	—	22	5	0	17	17	4	4	0	—	—	—	—	—	—
AMS	3	1	1	26	—	—	16	5	3	1	3	0	—	—	—	—
TZP	3	0	24	3	17	0	21	0	4	0	3	0	9	0	15	0
CFZ	1	3	7	20	0	17	16	5	3	1	0	3	1	8	19	0
CFE	—	—	26	1	1	16	18	3	3	1	2	1	—	—	—	—
CFN	—	—	10	17	0	17	17	4	3	1	2	1	—	—	—	—
CEFO	4	0	1	0	17	0	18	3	4	0	3	0	7	2	19	0
CEFZ	4	0	26	1	17	0	18	3	4	0	3	0	9	0	16	2
CEFX	4	0	1	0	17	0	18	3	4	0	3	0	8	0	19	0
CEFE	4	0	26	1	17	0	18	3	4	0	3	0	9	0	19	0
ATM	—	—	1	0	17	0	18	3	4	0	3	0	—	—	9	9
MER	4	0	1	0	17	0	21	0	4	0	3	0	9	0	19	0
AMI	2	0	27	0	17	0	21	0	4	0	3	0	9	0	19	0
GEN	4	0	27	0	16	1	20	1	3	1	3	0	9	0	19	0
CIP	3	1	26	1	17	0	18	3	3	1	3	0	8	1	18	1
NIT	—	—	0	1	14	3	15	6	1	3	0	3	0	1	—	—
TMP	3	0	16	11	9	8	10	11	0	4	2	1	3	5	16	2

a S, susceptible; R, resistant; —, not available; AMP, ampicillin; AMC, amoxicillin-clavulanic acid; AMS, ampicillin-sulbactam; TZP, piperacillin-tazobactam; CFZ, cefazolin; CFE, cefuroxime; CFN, cefoxitin; CEFO, cefotaxime; CEFZ, ceftazidime; CEFX, ceftriaxone; CEFE, cefepime; ATM, aztreonam; MER, meropenem; AMI, amikacin; GEN, gentamicin; CIP, ciprofloxacin; NIT, nitrofurantoin; TMP, trimethoprim-sulfamethoxazole.

The patterns of resistance for all eight bacteria are shown in Table 2. A minimum of 3 and a maximum of 13 antibiotic combinations were obtained for the MDR exploration. MDR is described as the resistance of a bacterial strain to at least one antimicrobial agent in three or more antimicrobial categories (7). Based on the results, *Aeromonas* spp., Enterobacter spp., K. pneumoniae, Proteus spp., and *Providencia* spp. showed resistance to at least three categories of antibiotics and therefore were regarded as MDR bacteria. Of the 5 MDR K. pneumoniae isolates, 3 isolates demonstrated resistance to 13 antibiotics and were found to be extended-spectrum β-lactamase (ESBL)-producing bacteria.

**TABLE 2:** Resistance patterns of bacterial isolates from poultry farms

AR profile^*a*^	No. of isolates (%)
Acinetobacter spp. (*n* = 4)	
AMS, CFZ	1 (25.0)
CFZ, CIP	1 (25.0)
*Aeromonas* spp. (*n* = 27)	
AMS, CFZ, CFN	10 (37.0)
AMS, CFZ, CFN, TMP	7 (25.9)
AMS, AMC, CFZ, TMP	2 (7.4)
Enterobacter spp. (*n* = 17)	
AMC, CFZ, CFE, CFN	7 (41.2)
AMC, CFZ, CFE, CFN, TMP	6 (35.3)
AMC, CFZ, CFE, CFN, NIT	3 (17.6)
K. pneumoniae (*n* = 21)	
AMP, TMP	6 (28.5)
AMP, AMS, CIP, NIT, TMP	2 (9.5)
AMP, AMC, AMS, CFZ, CFE, CFN, CEFO, CEFZ, CEFX, CEFE, ATM, NIT, TMP	3 (14.3)
Proteus spp. (*n* = 4)	
AMP, NIT, TMP	2 (50.0)
*Providencia* spp. (*n* = 3)	
AMP, CFZ, NIT	3 (100.0)
Pseudomonas spp. (*n* = 9)	
CFZ, TMP	5 (55.6)
S. paucimobilis (*n* = 19)	
CEFZ, CEFE	2 (10.5)

a AMP, ampicillin; AMC, amoxicillin-clavulanic acid; AMS, ampicillin-sulbactam; CFZ, cefazolin; CFE, cefuroxime; CFN, cefoxitin; CEFO, cefotaxime; CEFZ, ceftazidime; CEFX, ceftriaxone; CEFE, cefepime; ATM, aztreonam; AMI, amikacin; GEN, gentamicin; CIP, ciprofloxacin; NIT, nitrofurantoin; TMP, trimethoprim-sulfamethoxazole.

**FIG 1:**
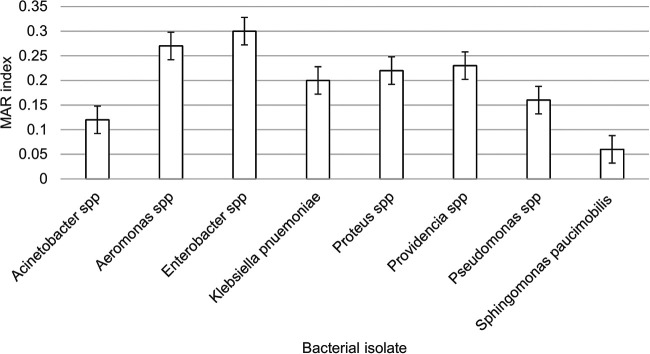
Distribution of MAR index values for isolated GNB.

Given that many isolates were known to acquire MAR, MAR index values were calculated to identify the possible high-risk source of ARB from the poultry farms. Figure 1 shows the distribution of MAR indices of the isolates. Bacterial species with a MAR index of ≥0.2 indicate a likelihood of origination from a high-contamination area, where antibiotics had been used extensively (6). Furthermore, the Kruskal-Wallis test showed that the bacteria exhibited significantly high percentage of AR and MAR index values (*P* < 0.0001).

**FIG 2:**
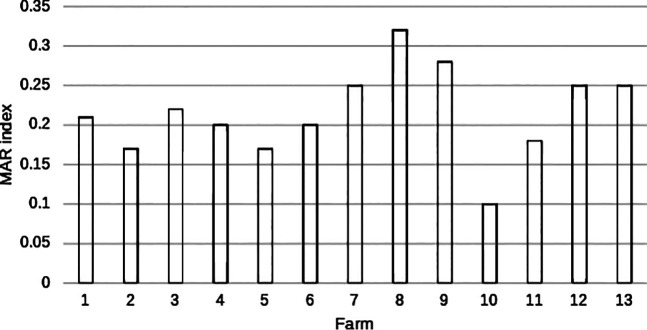
Distribution of MAR index values for the poultry farms.

A total of eight bacterial species were identified at each farm. Figure 2 illustrates the MAR index distribution for the farms. The ratios of the MAR index values indicated an insignificant difference in the MAR index values among the 13 poultry farms. Nine of the 13 farms had MAR index values of ≥0.2, and 5 farms which falls under ambiguity had MAR index values between 0.20 and 0.25.

In Malaysia, very limited studies have been conducted on various AR GNB in farm environments (8–12). Our study is the first to quantify the prevalence of eight types of MDR GNB isolated from the environment of poultry farms. K. pneumoniae is one of the most critical MDR bacteria, which has been unnoticed in veterinary and environmental health (13). To date, very limited data have been published on K. pneumoniae isolated from poultry environments around the world, and no data have been published locally. In Egypt, K. pneumoniae strains were recovered at 16.7% from poultry environmental samples, in which all of the isolates were MDR bacteria. In our study, the prevalence of K. pneumoniae isolated from environmental samples was 20.2% (21/204 isolates), with 23.8% (5/21 isolates) of the K. pneumoniae isolates being MDR (14, 15).

Klebsiella spp. are intrinsically resistant to penicillins and can acquire resistance to the third- and fourth-generations of cephalosporins by producing ESBL (9). Consistent with that statement, it was noted in our study that 100% of the isolates (21/21 isolates) exhibited resistance to ampicillin and 14% (3/21 isolates) showed resistance to monobactam (aztreonam) and third- and fourth-generation cephalosporins (cefotaxime, ceftazidime, ceftriaxone, and cefepime), labeled ESBL-producing strains.

Enterobacter spp. represent another potent zoonotic pathogen in poultry with very limited data (16, 17). Enterobacter spp. have been found to be intrinsically resistant to ampicillin, amoxicillin-clavulanic, and first-generation cephalosporins, including cefoxitin, but still susceptible to third- and fourth-generation cephalosporins (18, 19). In good agreement with these statements, 100% of Enterobacter isolates (17/17 isolates) in this study were resistant to amoxicillin-clavulanic acid, cefazolin, and cefoxitin, while 94.1% (16/17 isolates) and 47% (8/17 isolates) of Enterobacter isolates were resistant to cefuroxime and trimethoprim-sulfamethoxazole, respectively. All Enterobacter isolates were susceptible to third- and fourth-generation cephalosporins.

Additionally, the MAR index values of ≥0.2 for 9 of the 13 farms suggested the presence of MDR genes in bacteria from the environment (20). The majority of farms that used antibiotics were found to be significant sources of contamination. Nevertheless, 50% of the farms for which antibiotic usage was unclear also showed high MAR index values. Poor record management regarding antibiotic use was one of the challenges faced during sample collection and data analysis. In addition, the isolates from the respective farms, which vary in strain and the number of antibiotics tested, pose a challenge for categorization between high and low risk.

In conclusion, GNB isolated from the environment of poultry farms exhibited high rates of MDR against various antibiotics. The presence of these ARB is alarming because it threatens the effectiveness of available drugs and health care treatments to cure infectious diseases, turning them into life-threatening MDR diseases. Furthermore, poultry farmers, workers, and residents of nearby communities are at higher risk of exposure to ARB. Therefore, strict control of the use of antibiotics by regulatory authorities is crucial in managing the use of antibiotics in poultry industries to reduce the widespread emergence of ARB. Other proactive efforts, including further studies that address the One Health issues and determine the linkage of AR in animals, the environment, and humans, should be encouraged to combat and prevent the development and spread of ARB.

### Data availability.

The data set used and/or analyzed during the study are available from the corresponding author on reasonable request.
