# Supplementary material for: Multidrug-Resistant Gram-Negative Bacteria and Extended-Spectrum β-Lactamase-Producing Klebsiella pneumoniae from the Poultry Farm Environment
Source: Microbiol Spectr. 2022 Apr 25;10(3):e02694-21. doi: 10.1128/spectrum.02694-21 (PMC9241921; doi:10.1128/spectrum.02694-21)
Supplement: SUPPLEMENTAL FILE 1 — Supplemental material. Download spectrum.02694-21-s001.pdf, PDF file, 0.1 MB [file spectrum.02694-21-s001.pdf]

Supplementary Material

Table S1. Details on each Isolates based on selected farms

Farm	Sample	Isolate	Antibiotic resistance	Total antibiotic	Resistance percentage	MAR index	Usage of antibiotic
Farm 1	Soil	Aeromonas spp.	2	12	16.67	0.17	amoxicillin, neomycin, norfloxacin, lincomycin
	Soil	Aeromonas spp.	3	11	27.27	0.27	
	Soil	Enterobacter spp.	7	18	38.89	0.39	
	Soil	Enterobacter spp.	6	18	33.33	0.33	
	Soil	Enterobacter spp.	6	18	33.33	0.33	
	Soil	Klebsiella pneumoniae	1	19	5.26	0.05	
	Soil	Klebsiella pneumoniae	1	19	5.26	0.05	
	Soil	Klebsiella pneumoniae	2	19	10.53	0.11	
Farm 2	Water	Aeromonas spp.	1	12	8.33	0.08	amoxicillin
	Water	Aeromonas spp.	3	12	25	0.25	
	Water	Enterobacter spp.	6	17	35.29	0.35	
	Soil	Enterobacter spp.	7	18	38.89	0.39	
	Water	Klebsiella pneumoniae	3	20	15	0.15	
	Soil	Klebsiella pneumoniae	1	19	5.26	0.05	
	Water	Sphingomonas paucimobilis	0	12	0	0	
	Soil	Sphingomonas paucimobilis	1	11	9.09	0.09	
Farm3	Soil	Aeromonas spp.	3	12	25	0.25	not mentioned
	Soil	Aeromonas spp.	4	12	33.33	0.33	
	Water	Enterobacter spp.	7	18	38.89	0.39	
	Water	Klebsiella pneumoniae	1	19	5.26	0.05	
	Soil	Klebsiella pneumoniae	5	19	26.32	0.26	
	Soil	Klebsiella pneumoniae	5	19	26.32	0.26	
	Water	Providencia spp.	4	19	21.05	0.21	
	Water	Pseudomonas spp.	0	11	0	0	
Farm 4	Soil	Aeromonas spp.	3	12	25	0.25	used when needed but never specify

	Water	Aeromonas spp.	3	12	25	0.25	
	Soil	Enterobacter spp.	7	18	38.89	0.39	
	Soil	Klebsiella pneumoniae	1	19	5.26	0.05	
	Soil	Pseudomonas spp.	3	11	27.27	0.27	
	Soil	Pseudomonas spp.	2	11	18.18	0.18	
	Soil	Sphingomonas paucimobilis	0	11	0	0	
	Soil	Sphingomonas paucimobilis	2	12	16.67	0.17	
Farm 5	Water	Aeromonas spp.	3	12	25	0.25	not mentioned
	Water	Aeromonas spp.	4	12	33.33	0.33	
	Soil	Aeromonas spp.	4	12	33.33	0.33	
	Soil	Klebsiella pneumoniae	2	19	10.53	0.11	
	Water	Proteus spp.	2	19	10.53	0.11	
	Water	Providencia spp.	5	19	26.32	0.26	
	Soil	Sphingomonas paucimobilis	0	12	0	0	
	Soil	Sphingomonas paucimobilis	0	12	0	0	
Farm 6	Water	Aeromonas spp.	4	12	33.33	0.33	used when needed but never specify
	Water	Aeromonas spp.	2	12	16.67	0.17	
	Water	Enterobacter spp.	7	18	38.89	0.39	
	Water	Enterobacter spp.	8	18	44.44	0.44	
	Water	Sphingomonas paucimobilis	0	12	0	0	
	Soil	Sphingomonas paucimobilis	1	12	8.33	0.08	
	Soil	Sphingomonas paucimobilis	1	12	8.33	0.08	
	Soil	Sphingomonas paucimobilis	1	11	9.09	0.09	
Farm 7	Water	Aeromonas spp.	3	12	25	0.25	not mentioned
	Soil	Aeromonas spp.	4	12	33.33	0.33	
	Soil	Aeromonas spp.	5	12	41.67	0.42	
	Water	Enterobacter spp.	6	18	33.33	0.33	
	Water	Enterobacter spp.	6	18	33.33	0.33	
	Soil	Proteus spp.	7	20	35	0.35	
	Water	Sphingomonas paucimobilis	0	12	0	0	
	Water	Sphingomonas paucimobilis	0	10	0	0	

Farm 8	Water	Aeromonas spp.	2	12	16.67	0.17	not mentioned
	Water	Aeromonas spp.	8	20	40	0.4	
	Soil	Aeromonas spp.	5	12	41.67	0.42	
	Water	Enterobacter spp.	7	18	38.89	0.39	
	Water	Enterobacter spp.	7	18	38.89	0.39	
	Water	Enterobacter spp.	6	18	33.33	0.33	
	Soil	Proteus spp.	5	20	25	0.25	
	Soil	Sphingomonas paucimobilis	2	12	16.67	0.17	
Farm 9	Water	Aeromonas spp.	1	12	8.33	0.08	use as prophylactics but never specify
	Water	Aeromonas spp.	2	12	16.67	0.17	
	Soil	Aeromonas spp.	2	12	16.67	0.17	
	Water	Klebsiella pneumoniae	3	19	15.79	0.16	
	Soil	Klebsiella pneumoniae	15	20	75	0.75	
	Water	Klebsiella pneumoniae	16	21	76.19	0.76	
	Soil	Sphingomonas paucimobilis	0	12	0	0	
	Soil	Sphingomonas paucimobilis	2	11	18.18	0.18	
Farm 10	Water	Acinetobacter spp.	1	11	9.09	0.09	not mentioned
	Water	Acinetobacter spp.	2	10	20	0.2	
	Soil	Acinetobacter spp.	0	11	0	0	
	Soil	Klebsiella pneumoniae	1	19	5.26	0.05	
	Water	Klebsiella pneumoniae	1	19	5.26	0.05	
	Soil	Klebsiella pneumoniae	2	19	10.53	0.11	
	Water	Klebsiella pneumoniae	2	19	10.53	0.11	
	Water	Providencia spp.	4	19	21.05	0.21	
Farm 11	Water	Aeromonas spp.	1	11	9.09	0.09	not mentioned
	Water	Aeromonas spp.	3	12	25	0.25	
	Soil	Enterobacter spp.	6	18	33.33	0.33	
	Water	Proteus spp.	3	19	15.79	0.16	
	Water	Pseudomonas spp.	2	11	18.18	0.18	
	Water	Pseudomonas spp.	2	11	18.18	0.18	
	Water	Pseudomonas spp.	2	11	18.18	0.18	

	Soil	Sphingomonas paucimobilis	1	12	8.33	0.08	
Farm 12	Soil	Acinetobacter spp.	2	12	16.67	0.17	used when needed but never specify
	Soil	Aeromonas spp.	3	12	25	0.25	
	Water	Aeromonas spp.	4	11	36.36	0.36	
	Soil	Klebsiella pneumoniae	1	19	5.26	0.05	
	Soil	Klebsiella pneumoniae	15	20	75	0.75	
	Soil	Pseudomonas spp.	2	9	22.22	0.22	
	Soil	Pseudomonas spp.	1	11	9.09	0.09	
	Soil	Sphingomonas paucimobilis	1	12	8.33	0.08	
Farm 13	Water	Aeromonas spp.	8	12	66.67	0.67	used when needed but never specify
	Soil	Enterobacter spp.	6	18	33.33	0.33	
	Water	Enterobacter spp.	8	18	44.44	0.44	
	Water	Klebsiella pneumoniae	2	19	10.53	0.11	
	Water	Klebsiella pneumoniae	2	19	10.53	0.11	
	Soil	Pseudomonas spp.	2	11	18.18	0.18	
	Soil	Sphingomonas paucimobilis	1	12	8.33	0.08	
	Water	Sphingomonas paucimobilis	1	11	9.09	0.09	
